# The Use of Drones in Emergency Medicine: Practical and Legal Aspects

**DOI:** 10.1155/2019/3589792

**Published:** 2019-12-02

**Authors:** Anna Konert, Jacek Smereka, Lukasz Szarpak

**Affiliations:** ^1^Faculty of Law and Administration, Lazarski University, Swieradowska 43 str., 02-662 Warsaw, Poland; ^2^Department of Emergency Medical Service, Wroclaw Medical University, Parkowa 34, Wroclaw, Poland; ^3^Medical Faculty, Lazarski University, Swieradowska 43 str., 02-662 Warsaw, Poland

## Abstract

Unmanned aerial vehicles, also known as drones, can play a significant role in military and civil emergency medicine. The aim of the study was to present the real possibilities of using them in rescue operations and to provide examples from all over the world. Unmanned aerial vehicles can be applied to transport goods on demand, provide blood in urban areas, save sinking people, analyse the scale of damages, monitor large human gatherings, perform exploration activities, deliver blood samples and other analysis material, provide automated external defibrillators, support rescue operations and air transport, and perform agricultural activities. One must, however, be aware of the existing regulations regarding drone flights as an appearance of an unreported unmanned aircraft in the controlled space is identified worldwide as affecting aviation safety.

## 1. Introduction

Air transport is widely used in military and civil emergency medicine owing to the speed of action, lack of restrictions characteristic of ground vehicles, and the ability to reach distant, otherwise inaccessible places. However, apart from these undoubted benefits, air transport also has limitations, which include dependence on weather conditions, relatively lower load capacity compared with ground ambulances, and much higher costs than in the case of land transport.

Unmanned aerial vehicles, commonly known as drones, may be an alternative. The 20^th^ century saw the development of unmanned aerial vehicles controlled by radio waves, but it was the turn of the 20^th^ and 21^st^ centuries that resulted in an increased interest in their application for military purposes, mainly as an element of object recognition or antimissile exercises. Work on unmanned aircrafts for direct transport and medical evacuation has started recently [1]. Here, it is worth mentioning, among others, the idea of Black Knight Hummingbird by an American company Advanced Tactics Inc., or ILX-27 developed by the Institute of Aviation in cooperation with Military Aviation Works No. 1 in Lodz, Poland.

These machines, in spite of their relatively small dimensions, are capable of carrying up to 5000 kg of cargo, including people and weaponry. In battlefield conditions, they can effectively replace medevac helicopters in the area of evacuation of the wounded [2, 3].

The aim of the study is to present the real possibilities of using unmanned aerial vehicles in rescue operations through an analysis of examples.

## 2. World Applications of Drones in Rescue Operations

The opening of the aviation market to the civil use of unmanned aircrafts has started a new era for aviation [4]. Today, anyone can buy a drone which can fly in the range of 5–7 km, up to several hundred meters or even higher. The vision of transporting goods on demand, providing blood in urban areas, or saving sinking people is becoming reality [5, 6]. Amukele et al. analysed the quality of red blood cell units, apheresis platelet units, and unthawed plasma units frozen within 24 hours of collection placed in a cooler attached to the drone and flown for up to 26.5 minutes with ambient temperatures ranged between –1 and 18°C. They revealed that there was no adverse impact of drone transport and no evidence of red blood cell haemolysis; no significant changes in platelet count, pH, and other blood parameters limiting the possibility of using blood products [5]. This study suggested that the drone could be a good option for the transportation of blood products [5].

Significantly, the rapid development of unmanned aircrafts and their operations is also possible because, contrary to manned aviation, in some countries there were no regulations regarding the design, production, or rules for the use of unmanned aircrafts [7]. However, in many European countries there is a strict legislation in where according to the class of the drone (which is mainly related to its weight) different restrictions exist. For drones heavier than a certain limit, the operators must acquire an appropriate license before even flying a drone. A flight plan must be also submitted for the heavier ones, while certain flight height/place restrictions also exist (drones cannot fly near airports, above crowd, etc.). The legal aspects have an impact on development and testing of new technologies and on the use of unmanned aviation on a broader scale.

The United Nations Institute for Training and Research (UNITAR) was one of the pioneers in the use of new technologies in the United Nations. The UNITAR Operational Satellite Applications Programme (UNOSAT) aims to facilitate change in key areas such as people's security and humanitarian aid. The first use of drones under this programme took place in 2010 in Haiti to analyse the damage caused by earthquake [7]. Drones are also used to monitor large human gatherings. An example was monitoring the Paléo Festival in Nyon in 2012 with the use of unmanned aerial vehicles [6]. The applied drones floated in the air over the gathered people for up to 20 minutes; the transmission of the image to the operator allowed for a quick response to threats.

Drones can be used even in cloudy conditions thanks to their low flight altitude, which could be difficult or completely impossible with normal helicopters. Another advantage of drone monitoring is the fact that the recording from the cameras can be played back again, so the video content can be viewed by several people. This minimizes the risk of overlooking important details and is of particular importance.

Scientific literature indicates numerous applications of unmanned aerial vehicles during exploration activities. An example is the use of thermal imaging cameras by the Canadian police. The action ended with locating a man in a car wreck in a desert area, thanks to which the rescue services could reach the victim more quickly.

Drones are also used as means of transport. One of the first examples was in 2015, when search and rescue services delivered life jackets to people trapped on rocks in the middle of the Little Androscoggin River in the state of Maine. The water rescue services in Chile and Iran are equipped with drones with a lifebelt, as well as audio and video tools to communicate with the person to be rescued.

Two hospitals in Switzerland started using drones to deliver blood samples and other analysis material. This is the first case of a commercial application of unmanned aerial vehicles for medical purposes, giving hope for saving peoples' lives faster than nowadays. These drones can carry light loads, of less than 2 kg, and move at speeds of up to 36 km/h; their maximum range is 20 km. In case of a breakdown, the machines are also equipped with parachutes so they can safely fall to the ground surface without destroying the delicate cargo [8].

The first drone which already performs commercial transportation flights is MD4-100 from a German company MicroDrones, in the fleet of DHL. This unmanned aircraft in the airspace of the German Air Control Service (DFS) transports drugs several times a week between the German island of Juist (North Sea) and Norddeich, a city located in the north of Germany [2].

The Karolinska Institute in Sweden connected a defibrillator to a drone owned by a fire station in Norrtälje, located in rural areas near Stockholm, and then sent it to a place at a distance of 10 km where there were reports of heart attacks in the last 8 years. It turned out that the average time from 18 flights of the drone was 5 minutes and 21 seconds compared with 22 minutes when using an ambulance [9, 10]. An unmanned aerial vehicle with an automatic defibrillator can move at a speed of about 100 km/h, which translates into faster defibrillation and increases the chances of spontaneous circulation return. It is important that the drone communicates with the person in charge of the rescue operation so the automated external defibrillator is delivered as close to the victim as possible.

A study published by Bogle et al. described drone-based AED deployment networks. The authors revealed that all analysed drone networks improved AED delivery time and suggested that they can improve out-of-hospital cardiac arrest survival outcomes [11].

Currently, work is in progress on the international and regional levels (European Union regulation) to establish common rules on using drones. A new European Union regulation on remotely piloted aircraft systems (RPAS) will enter into force in the coming months. The legislative process that aims at the extension of the European Union competence to include safety regulations in this area is ongoing. On September 11, 2018, the revised basic regulation [12] has entered into force. This might facilitate the movement of drone operators within the European Union.

Enabling the operation of remotely controlled aircrafts or even the application of automated flight control systems (as part of the so-called autonomous flights) requires the development of detailed solutions for a number of technical and operational aspects, which include, inter alia, the following issues. First of all, the issue of airworthiness, i.e., rules of design, production, release to service, and maintenance of the devices, including ground control stations of unmanned aircrafts. Secondly, data transmission from a ground control station of unmanned aircrafts. This includes ensuring continuity of data transmission, protection from unauthorized interference, available radio frequencies, or other data transmission techniques. Moreover, the available radio frequencies for data transmission systems, motion detection and collision avoidance systems, emergency systems (e.g., loss of communication), unmanned aircraft operators including training and competence requirements, and the rules of the air including procedures for air traffic services [13].

Smolyanskiy et al. presented a micro-aerial vehicle (MAV) system, built with inexpensive off-the-shelf hardware, for autonomously following trails in unstructured, outdoor environments. This system is based on a deep neural network enabling view orientation and lateral offset of the MAV. This system is able to navigate forest trails more robustly than previous techniques, including autonomous flights of 1 km. This construction is an important stage in the development of drones towards more autonomous devices, especially in difficult terrain, such as flying on the forest land [14].

Loquercio et al. in contrast to traditional “map-localize-plan” methods, propose DroNet: a neural network that can safely drive a drone through the streets of a city. This system allows drones to successfully fly at relative high altitudes and even in indoor environments, such as parking lots and corridors [15].

Palossi et al. analysed autonomous unmanned aerial vehicles (UAVs) with advanced computer vision techniques based on computationally expensive algorithms, to navigate in difficult environments. Taking into account that autonomous flight is considered unaffordable in the context of nanoscale UAVs, they have presented the first vertically integrated system for fully autonomous deep neural network-based navigation on nanosize UAVs [16].

Passalis and Tefas proposed a deep reinforcement learning method for continuous fine-grained drone control that allows for acquiring high-quality frontal view person shots for various aerial cinematography tasks. They have demonstrated that the proposed method can be also effectively combined with simulation environments that support only discrete control commands, improving the control accuracy. Such technological solutions may also be used in the case of drones used for emergency purposes, including medical use [17].

Drafting regulations that would enable the operation and development of unmanned aircrafts is therefore an extremely difficult task. The regulations must assume the necessity of integrating unmanned and manned aviation. Achieving this integration requires, first and foremost, a further development of technology whereby the level of safety of unmanned flight operations will be at least the same as that achieved in manned aviation. The availability of proven technological or organizational solutions will, as a result, allow for the development of legislations that will enable a wider use of unmanned aviation and the safe integration with manned aviation [17].

It is very important for the usage of drones for medical purposes that law allows for the so-called beyond visual line of sight (BVLOS) operations. In most countries, it is now possible to operate with the visual line of sight (VLOS). BVLOS operations, usually more complex, still remain not possible in most countries, and in others they are allowed under strict conditions. Just recently, in Poland, a new regulation allowing BVLOS operations has been published. Poland is thus among the first European countries that created special regulations in this area.

## 3. Using Drones for Medicine—The Polish Perspective

The Polish Air Force Institute of Technology created a drone called AtraxM, designed to support rescue operations and air transport. The drone can perfectly identify the place of the accident, the number of victims, and the scale of the event before the arrival of emergency services. For medical purposes, AtraxM has been equipped with replaceable trays for transferring first aid kits containing basic dressing materials, life-saving shock-recovery kits (when there is a person entitled to administer medicines like a doctor or nurse at the place of the event), medicines directly prepared for the patient's need for immediate administration (e.g., ampoules with adrenaline and insulin) when symptoms are known and a quick reaction is required, medicines for people living in areas affected by natural disasters, e.g., floods, and blood bags [5, 18].

The institute is working on the development of AtraxM; the goal is to include a container for the first diagnosis with such medical devices as ECG, glucometer, devices for measuring blood pressure and temperature (optional), with data sent directly to the rescuer-dispatcher, who will give further instructions on how to proceed; and an automated external defibrillator for safe defibrillation in the event of cardiac arrest and a container with an oxygen concentrator or nebulizer for quick reaction in situations of respiratory failure [18].

Another Polish project, called AirVein, assumes the creation of a system that will help hospitals and Regional Centres of Blood Donation and Blood Treatment in a quicker exchange of blood. The goal is to create a fleet of unmanned aerial vehicles, terrestrial infrastructure equipped with a hybrid navigation system, modules for storing and moving blood while maintaining the required parameters, and an information technology system coordinating the delivery [19, 20].

Regarding the legislation, Poland was one of the first European countries which created the possibility to fly with drones (VLOS operation). The Aviation Law Act of July 3, 2002 [21], in its art. 126, paragraph 1, states that unmanned aerial vehicles may be operated in the Polish airspace. Paragraph 2 introduces the obligation for unmanned aerial vehicles to be equipped with the same flight, navigation, and communication facilities as a manned aircraft with regard to the visual (VFR) or instrument flight rules (IFR) within a defined class of airspace. In accordance with the regulations, unmanned flights are allowed provided that certain requirements for the equipment and the qualifications of the flight crew are met. Pursuant to the act, the detailed conditions and rules for the operation of unmanned flights have been specified in the relevant regulations [22].

The Regulation of the Minister of Transport, Construction and Maritime Economy of March 26, 2013, on the exclusion of certain provisions of the Aviation Law Act—for certain types of aircraft and the specification of conditions and requirements for the use of these aircrafts [23] was the very first attempt to regulate the general requirements for unmanned aircraft operations and was among the first regulations in Europe. It creates detailed rules on flights, operator responsibility, etc.; it was amended in 2016 and 2018. The regulation allows only VLOS operations and contains its definition—i.e., operations in which the operator or observer of the flying model maintains a direct eye contact with the flying unmanned aircraft.

As for BVLOS operations, the Aviation Law allows them to be conducted only in prior segregated airspace, and the operator must file a motion to the Polish Air Navigation Services Agency (PANSA).

On January 17, 2019, a new Regulation was published and entered into force, amending the 2016 Regulation, introducing some changes for VLOS operations, and creating new rules for BVLOS operations in order to facilitate them [24]. First of all, there will be an obligation to register drones used for BVLOS via an entry in the register of files in the Civil Aviation Authority (CAA). Secondly, BVLOS flights will be allowed for drones weighing up to 25 kg. They must be equipped with anticollision and precision lighting as on a manned aircraft, with a camera to observe the surroundings and with devices that automatically maintain altitude and distance from the operator below the maximum allowable value, monitor flight parameters, enable location of the basic drone (location, speed, altitude, and direction of flight), and emergency location in the case of communication loss or control possibilities. Moreover, such drones must be able to automatically perform emergency procedures: (1) continue the flight along the programmed route; (2) end the flight by emergency landing; and (3) make a flight to the preprogrammed location. The most important change from the point of view of the subject matter of this article is that BVLOS flights will be allowed outside the dedicated zone in the case of the following flights: operational (for the needs of police, border guards, firefighting, health, search and rescue services, and protection of state security); specialized (for the purposes of supervision, monitoring, control or protection, surveying, and agriculture or forestry); automatic (for the needs of supervision, monitoring, control and protection, and agrotechnical activities); and training. It is therefore possible now to use drones for medical purposes with a takeoff mass less than 25 kg. But the entity would need to obtain the consent of the President of CAA for such a flight; also, an air traffic service provider needs to publish information on the planned flights and to be informed by the entity at least 7 days before the flight dates. Operational, specialist, and training flights can be performed up to 120 m above ground level and with visibility not less than 5 km. The flights will be carried out by a 2-man crew.

Finally, the term “automatic flight” has been defined in the regulation as a flight on a programmed route without the participation of an operator, but with the capability to immediately take over the remote control. Automatic flights can be performed up to 50 m above ground level or up to the height of 10 m above the highest obstacle within a radius of 100 m from the place of flights and at a horizontal distance of at least 150 m from settlements and other population centres.

The creation of such rules not only enables the technical development of unmanned systems in Poland but also helps different entities to do their job better and quicker. This refers to the police, border guards, firefighting, health, search and rescue teams, etc. Finally, there is a hope to save more peoples' lives quicker. Only 1 of 10 people survive cardiac arrest outside a hospital. Each minute without defibrillation increases the risk of death by 10% [25]. Thus, 5 minutes compared with 20 minutes can make a big difference in saving lives [1].

## 4. Conclusions

The use of drones for medical purposes brings many advantages, such as quick help, shortening the time of traveling to the patient, reduction of complications in the injured owing to a short time to wait for rescue, support and improvement of basic operations of medical emergency teams, and the opportunity to reach places inaccessible for basic means of medical transport (e.g., because of floods and blocked roads) [1].

It is very important, however, to be aware of the existing regulations. There are several safety information campaigns but neither information campaigns nor the most perfect regulations will protect against threats that may be caused by the presence of a drone in a place not intended for. An appearance of an unreported unmanned aircraft in the controlled space is an issue identified worldwide as affecting aviation safety. Examples may be filming a large passenger aircraft from a close distance, or an interruption of approach to an international airport associated with an identification of a drone [26].

## References

[1] Claesson A., Svensson L., Nordberg P. (2017). Drones may be used to save lives in out of hospital cardiac arrest due to drowning. *Resuscitation*.

[2] Balasingam M. (2017). Drones in medicine—the rise of the machines. *International Journal of Clinical Practice*.

[3] European Commission (2014). *Communication from the Commission to the European Parliament and the Council. A New Era for Aviation*.

[4] Van de Voorde P., Gautama S., Momont A., Ionescu C. M., De Paepe P., Fraeyman N. (2017). The drone ambulance [A-UAS]: golden bullet or just a blank?. *Resuscitation*.

[5] Amukele T., Ness P. M., Tobian A. A., Boyd J., Street J. (2017). Drone transportation of blood products. *Transfusion*.

[6] Sachan D. (2016). The age of drones: what might it mean for health?. *The Lancet*.

[7] Van Berlaer G., Staes T., Danschutter D. (2017). Disaster preparedness and response improvement: comparison of the 2010 Haiti earthquake-related diagnoses with baseline medical data. *European Journal of Emergency Medicine*.

[8] Mulero-Pázmány M., Jenni-Eiermann S., Strebel N., Sattler T., Negro J. J., Tablado Z. (2017). Unmanned aircraft systems as a new source of disturbance for wildlife: a systematic review. *PLoS One*.

[9] Rosser J. B., Parker B. C., Vignesh V. (2018). Medical applications of drones for disaster relief: a review of the literature. *Surgical Technology International*.

[10] Braun J., Gertz S. D., Furer A. (2019). The promising future of drones in prehospital medical care and its application to battlefield medicine. *Journal of Trauma and Acute Care Surgery*.

[11] Bogle B. M., Rosamond W. D., Snyder K. T., Zègre-Hemsey J. K. (2019). The case for droneassisted emergency response to cardiac arrest: an optimized statewide deployment approach. *North Carolina Medical Journal*.

[12] European Parliament (2018). *Regulation (EU) 2018/1139 of the European Parliament and of the Council of 4 July 2018 on Common Rules in the Field of Civil Aviation and Establishing a European Union Aviation Safety Agency, and Amending Regulations (EC) No 2111/2005, (EC) No 1008/2008, (EU) No 996/2010, (EU) No 376/2014 and Directives 2014/30/EU and 2014/53/EU of the European Parliament and of the Council, and Repealing Regulations (EC) No 552/2004 and (EC) No 216/2008 of the European Parliament and of the Council and Council Regulation (EEC) No 3922/91*.

[13] International Civil Aviation Organization (2011). *Unmanned Aircraft Systems (UAS)*.

[14] Smolyanskiy N., Kamenev A., Smith J., Birchfield S. Toward low-flying autonomous MAV trail navigation using deep neural networks for environmental awareness.

[15] Loquercio A., Maqueda A. I., del-Blanco C. R., Scaramuzza D. (2018). Dronet: learning to fly by driving. *IEEE Robotics and Automation Letters*.

[16] Palossi D., Loquercio A., Conti F., Flamand E., Scaramuzza D. Ultra low power deep-learning-powered autonomous nano drones.

[17] Passalis N., Tefas A. (2019). Continuous drone control using deep reinforcement learning for frontal view person shooting. *Neural Computing and Applications*.

[18] Amukele T. K., Hernandez J., Snozek C. L. H. (2017). Drone transport of chemistry and hematology samples over long distances. *American Journal of Clinical Pathology*.

[19] Glauser W. (2018). Blood-delivering drones saving lives in Africa and maybe soon in Canada. *Canadian Medical Association Journal*.

[20] Robakowska M., Ślęzak D., Tyrańska-Fobke A. (2019). Operational and financial considerations of using drones for medical support of mass events in Poland. *Disaster Medicine and Public Health Preparedness*.

[21] (2002). *Act of 3 July 2002 Aviation Law, Journal of Laws 2002, no. 130, item 1112, with Amendments*.

[22] Konert A., Kotliński M. (2018). Polish regulations on unmanned aerial vehicles. *Transportation Research Procedia*.

[23] (2016). *Act of 8 August 2016, Aviation Law, Journal of Laws 2016, no. 130, item 1317, with Amendments*.

[24] Mesar T., Lessig A., King D. R. (2018). Use of drone technology for delivery of medical supplies during prolonged field care. *Journal of Special Operations Medicine*.

[25] Lippi G., Mattiuzzi C. (2016). Biological samples transportation by drones: ready for prime time?. *Annals of Translational Medicine*.

[26] Konert A., Kotliński M. (2018). “How come I cannot fly a drone above the Prime Minister’s office?”—criminal and civil liability of a drone operator in Poland. *Ius Novum*.

